# Reversible dual inhibitor against G9a and DNMT1 improves human iPSC derivation enhancing MET and facilitating transcription factor engagement to the genome

**DOI:** 10.1371/journal.pone.0190275

**Published:** 2017-12-27

**Authors:** Juan Roberto Rodriguez-Madoz, Edurne San Jose-Eneriz, Obdulia Rabal, Natalia Zapata-Linares, Estibaliz Miranda, Saray Rodriguez, Angelo Porciuncula, Amaia Vilas-Zornoza, Leire Garate, Victor Segura, Elizabeth Guruceaga, Xabier Agirre, Julen Oyarzabal, Felipe Prosper

**Affiliations:** 1 Cell Therapy Program, Center for Applied Medical Research (CIMA), University of Navarra, Pamplona, Spain; 2 Oncohematology Program, Center for Applied Medical Research (CIMA), University of Navarra, Pamplona, Spain; 3 Small Molecule Discovery Platform, Molecular Therapeutics Program, Center for Applied Medical Research (CIMA), University of Navarra, Pamplona, Spain; 4 Bioinformatics Unit, Center for Applied Medical Research (CIMA), University of Navarra, Pamplona, Spain; 5 Hematology and Area of Cell Therapy, Clínica Universidad de Navarra, University of Navarra, Pamplona, Spain; University of Texas at Austin Dell Medical School, UNITED STATES

## Abstract

The combination of defined factors with small molecules targeting epigenetic factors is a strategy that has been shown to enhance optimal derivation of iPSCs and could be used for disease modelling, high throughput screenings and/or regenerative medicine applications. In this study, we showed that a new first-in-class reversible dual G9a/DNMT1 inhibitor compound (CM272) improves the efficiency of human cell reprogramming and iPSC generation from primary cells of healthy donors and patient samples, using both integrative and non-integrative methods. Moreover, CM272 facilitates the generation of human iPSC with only two factors allowing the removal of the most potent oncogenic factor cMYC. Furthermore, we demonstrated that mechanistically, treatment with CM272 induces heterochromatin relaxation, facilitates the engagement of OCT4 and SOX2 transcription factors to OSKM refractory binding regions that are required for iPSC establishment, and enhances mesenchymal to epithelial transition during the early phase of cell reprogramming. Thus, the use of this new G9a/DNMT reversible dual inhibitor compound may represent an interesting alternative for improving cell reprogramming and human iPSC derivation for many different applications while providing interesting insights into reprogramming mechanisms.

## Introduction

Induced pluripotent stem cells (iPSC) can be generated by overexpression of core pluripotency factors [1] and represent an important tool for studying development and regulatory mechanisms underlying pluripotency. Moreover, there is an unquestionable therapeutic potential of iPSCs for regenerative medicine [2]. However, the low efficiency of iPSC generation is a significant handicap for mechanistic studies, high throughput screenings, disease modelling and especially for therapeutic applications. In recent years, there has been a concerted effort to identify agents that enhance iPSC derivation, and compounds involved in epigenetic regulation, cell-signaling and survival have been reported to improve iPSC derivation [3,4]. Understanding the molecular mechanisms that underlie reprogramming to pluripotency is crucial for the development of optimized protocols for iPSC derivation.

Recent studies have enabled large-scale genomic, epigenomic and proteomic profiling of cells as they acquire pluripotency through the reprogramming process [5–7], revealing the dynamic nature of induced pluripotency and identifying ten major dynamic expression patterns [8]. Those studies have shown a multistep process that starts with the initial binding of OSKM factors to defined promoters and enhancer regions of the genome, that induces a highly proliferative state and the loss of somatic identity by an early mesenchymal to epithelial transition (MET) [9,10]. Interestingly, during this early phase there are large chromatin domains that are refractory to initial OSKM binding, called differentially bound regions (DBRs) [9], that contain genes required for pluripotency (such as NANOG, DPPA4 and GDF3). DBRs overlapped with lamin-associated domains (LADs) and were enriched in the heterochromatin marks H3K9me2 and H3K9me3 [9]. This binding impediment is overcome at later steps of reprogramming, although further work is required to understand the steps that allow binding of the transcription factors. The initial phase of reprogramming is followed by the transient expression of programs with early-developmental and pre-implantation-like characteristics, that finally promotes entry in the late reprogramming stage and the re-activation of pluripotency [8]. This maturation and stabilization phase is characterized by the activation of the core pluripotency circuitry and the silencing of the exogenous transgenes.

Gene expression changes during the reprogramming process are accompanied by removal of epigenetic roadblocks including histone and DNA methylation marks [11]. As example, regions that gained H3K4me2 at early time points of reprogramming, frequently coincided with OCT4 and SOX2 binding sites [8], which is consistent with a dominant role for these transgenes in chromatin remodelling at early stages of cell reprogramming. Consequently, small molecules targeting epigenetic regulators implicated in histone and/or DNA modifications have been described to play a key role as modulators of cell reprogramming [12,13], and have been reported to increase reprogramming capacity in both mouse and human iPSCs derivation [14–16]. In particular H3K9 methylation is a primary epigenetic determinant for the intermediate pre-iPSC state, and its removal leads to fully reprogrammed iPSCs [17,18]. BIX-01294 compound, a modulator of the G9a histone methyltransferase [19] that is responsible for mono- and di-methylation of H3K9 [20], was reported to enhance mouse iPSC generation in combination with DNA methyl transferase (DNMT) inhibitors [15,16]. Moreover BIX-01294 together with RG108, a DMNT inhibitor, or BayK, an L-calcium channel agonist, allowed mouse iPSC generation in the absence of *Sox2* and *cMyc*. However, their effect in human cells and the mechanistic changes in the reprograming roadmap beyond G9a and DNMTs inhibition that enable improved iPSC derivation were not clearly investigated.

We have recently developed novel substrate-competitive dual and reversible inhibitors against methyltransferase activity of G9a and DNMT1, with potent *in vitro* and *in vivo* activity [21]. Thus, we hypothesize that by inhibiting the repressive activities of G9a and DNMT1 with leading compounds, we could achieve relaxation of chromatin which could lead to improving the reprogramming process in human cells. Indeed, in this work we have demonstrated that treatment with CM272 compound improves iPSC derivation from both healthy donor and patient samples. We have also investigated the underlying mechanisms by which the new reversible dual G9a/DNMT inhibitor increases the efficiency of conversion to iPSC, that resulted in a general chromatin relaxation that facilitates the engagement of transcription factors to the genome at refractory binding regions and improves the early events during cell reprogramming of somatic cells to iPSCs.

## Materials and methods

### Small molecule compounds

CM272 and CM579 compounds (purity >95%) was synthesized as described [21]. BIX-01294 was purchased from Sigma-Aldrich (purity >98%).

### Cell culture

BJ human fibroblast cells (ATCC, CRL-2522) and 293T cells (ATCC, CRL-3216) were cultured in DMEM high glucose (Sigma) supplemented with 10% FBS (Gibco), 2 mM L-glutamine (Lonza), 1% NEAA (Lonza) and 100 UI/ml Penicillin/Streptomycin (Lonza). Adipose Derived Stem Cells (ADSCs) were obtained from the stromal vascular phases of adipose tissue from an adult donor as described [22]. Briefly, adipose tissue was carefully separated from skin and vessels, minced until getting a semi-solid paste and digested with 2 mg/mL of collagenase type I (Gibco). The lower phase containing the mesenchymal stem cells was filtered and seeded in gelatin-coated culture plates in Alpha Minimum Essential Medium supplemented with 10% FBS (Gibco), 2 mM L-glutamine (Lonza), 100 UI/ml Penicillin/Streptomycin (Lonza) and 1ng/mL of bFGF (Peprotech). ADSCs were expanded for a maximum of five passages before use. Human fibroblasts were obtained from a skin biopsy of healthy donors or primary hyperoxaluria type I patients as described [23]. Fibroblasts were cultured in gelatin-coated culture plates, for a maximum of five passages before reprogramming, in Dulbecco’s Modified Eagle Medium (DMEM, Sigma) supplemented with 10% FBS (Gibco), 1% NEAA (Lonza), 2 mM L-glutamine (Lonza), 100 UI/ml Penicillin/Streptomycin (Lonza) and 5 ng/ml bFGF (Peprotech). ADSCs and primary fibroblast were obtained after written informed consent. All cell lines were tested for mycoplasma.

### Virus production

Doxycycline inducible lentiviral vectors (FUW-Tet-O) coding for the human POU5F1 (OCT4), SOX2, KLF4 and cMYC transcription factors and the constitutive reverse tetracycline trans-activator lentiviral vector FUW-M2rtTA were obtained from Addgene (plasmids #20726, #20724, #20725, #20723 and #20342) and VSVG-coated lentiviruses were generated in 293T cells as described [24]. Briefly, 5x10^6^ 293T cells were seeded in 150cm^2^ culture dish 48h before transduction. Cells were transfected with FUW-TetO lentiviral vectors along with packaging plasmids psPAX2 and pMD2.G (Addgene plasmids #12260 and #12259), using Lipofectamine2000 transfection reagent according to the manufacturer’s recommendations. Virus-containing supernatants were collected 72h post-transfection, filtered through a 0.45 μm filter, concentrated by ultracentrifugation and quick-freeze in liquid nitrogen for long term storage.

### iPSC generation and culture

Human iPSCs using doxycycline-inducible lentiviral system were generated as described previously [24]. Briefly, human BJ fibroblasts, ADSCs and dermal primary fibroblasts (10^6^) were seeded 24h before transduction. Virus-containing supernatants were pooled for the indicated factors and supplemented with FUW-M2rtTA in a ratio 2:1 in fresh culture medium. Two consecutive infections were performed over a period of 48h in the presence of 4 μg/ml of polybrene. Two days after the last infection media was replaced by new culture media containing the small molecule compound at the indicated concentration and incubated for another 48h. Five days after the first infection (day 0), fibroblasts were passaged and replated by triplicate at different densities on MEF feeder layers. To induce reprogramming, culture medium was supplemented with 1 μg/ml doxycycline (Clontech) and 48h later fibroblast media was replaced by human iPSC culture media supplemented with 1 μg/ml doxycycline. For iPSC generation using non-integrative vectors Epi5 Episomal iPSC Reprogramming Kit (Invitrogen) and CytoTune-iPS 2.0 Sendai Reprogramming Kit (Invitrogen) were used according to manufacturer’s instructions. CM272 was added to the culture media at the indicated times and doses. iPSC colonies were identified based on ES cell–like morphology and alkaline phosphatase staining at day 30 of reprogramming. To establish iPSC lines, colonies were picked manually based on morphology between 4 and 8 weeks after doxycycline induction and maintained in the absence of doxycycline.

iPSCs were maintained under standard iPSC culture conditions on irradiated mouse embryonic fibroblast and were cultured in KO-DMEM (Gibco) containing 4.5 g/L glucose and supplemented with 20% knockout serum replacement (Gibco), 2 mM glutamine (Lonza), 1% NEAA (Lonza), 100 UI/ml Penicillin/Streptomycin (Lonza), 0.1 mM b-mercaptoethanol (Gibco), and 5 ng/ml bFGF (Peprotech). iPSCs were mechanically passaged every 5–7 days in the presence of 10 μM of ROCK1 inhibitor compound GSK269962A (AxonMedChem). Prior to teratoma formation iPSC were cultured on 2% Matrigel Basement Membrane Matrix (BD Biosciences) coated plates with conditioned iPSC media. For Tri-lineage differentiation iPSCs were cultured on Matrigel Basement Membrane Matrix (BD Biosciences) with mTeSR1 feeder-free cell culture medium (StemCell Technologies) according to the manufacturer’s instructions. All cell lines were tested for mycoplasma.

Reprogramming efficiency was calculated as the number of AP^+^ colonies formed per 10^5^ infected cells seeded.

### Trilineage differentiation of human iPSC and teratoma formation

Trilineage differentiation of generated iPSCs was performed using STEMdiff Trilineage Differentiation Kit (StemCell Technologies) according to the manufacturer’s instructions. This kit provides a monolayer culture assay to functionally validate the ability of iPSC lines to differentiate to the three germ layers: ectoderm, mesoderm, and endoderm. For teratoma formation, iPSCs grown in Matrigel Basement Membrane Matrix (BD Biosciences) were collected by collagenase IV treatment and subcutaneously injected into the dorsal flanks of 4–6 week-old male immuno-deficient Rag2^-/-^γc^-/-^ mice. Tumors generally developed within 5–8 weeks after injection and animals were sacrificed before tumor size exceeded 1.5 cm in diameter. Tumor nodules were dissected and fixed with 10% formalin (Sigma), embedded into paraffin and sectioned for hematoxylin/eosin staining. The presence of differentiated cells or tissues representative of the three embryonic germ layers was analyzed.

### Alkaline phosphatase staining and immunofluorescence

AP staining was performed using the Alkaline Phosphatase Detection Kit (Sigma) according to the manufacturer’s instructions. For immunofluorescence assay, human iPSCs were adhered on slide chambers (Nunc) containing MEFs, fixed with 4% paraformaldehyde (Sigma) for 30 minutes at RT and permeabilized for 10 minutes with 1% TritonX-100 (Sigma). Cells were blocked with 5% BSA for 30 minutes at RT and incubated for one hour at RT or o/n at 4°C with NANOG antibody (Abcam), SOX2 (R&D), SSEA-1 (Chemicon), SSEA-4 (Chemicon), TRA1-60 (Chemicon) and TRA1-81 (Chemicon) diluted in PBS/TBS with 1% BSA. FITC- or Cy3-conjugated secondary antibodies (Sigma) diluted in the same solution was incubated for 1–1.5 hours at RT. Nuclei were counterstained with 1:4 dilution of DAPI mounting medium (Vector Labs). Samples were visualized under an inverted fluorescence microscope.

### gDNA and RNA isolation, and quantitative PCR analysis

Genomic DNA was extracted using the Nucleo Spin Tissue DNA extraction kit (Macherey-Nagel) and total RNA was isolated using Trizol Reagent (Invitrogen) or Maxwell 16 LEV simplyRNA Tissue Kit (Promega) using a Maxwell 16 Research Instrument (Promega) according to the manufacturer's instructions. RNA quality was tested using Bioanalyzer (Agilent). cDNA was synthesized using PrimeScript RT reagent Kit (Takara) according to the manufacturer's instructions. cDNAs and DNAs from ChIP were amplified with SYBR Green qPCR mix (Applied Biosystems) in a 7300 Real-Time PCR System (Applied Biosystems). Oligonucleotide sequences are listed in S6 Table. Ct values used in our analysis corresponded to three PCR replicates and relative expression was analyzed by -ΔΔCt method. GAPDH expression was used for normalization. The enrichment of ChIP DNA over input DNA was calculated by enrichment = 2^(Ct ChIP DNA–Ct input DNA)^.

### Proliferation assay, Dot blot and cell cycle analysis

Cell proliferation was analyzed using the CellTiter 96 Aqueous One Solution Cell Proliferation Assay (Promega) according to the manufacturer’s instructions. Briefly, BJ cells were seeded in triplicate at a density of 5000 cells/well in 96-well plates. Twelve hours after cell seeding culture media was replaced by new media containing serial dilutions of CM272 or CM579 compound starting from 10μM. After 48h of treatment culture media was removed and cells were incubated for 1–2 hours with 100ul/well of RPMI1640 (Lonza) containing 20% FBS (Gibco) and 20ul/well of CellTiter 96 Aqueous One Solution reagent. Absorbance was measured at 490nm in a 96-well plate reader. Proliferation rate was calculated as the percentage of total absorbance of treated cells/absorbance of non-treated cells. The GI_50_ value of compound was determined using non-linear regression plots with the GraphPad Prism v5 software.

Dot Blot assay was performed as described [21]. Briefly, 500ng of genomic DNA was loaded onto a nitrocellulose membrane (GE Healthcare), previously wetted in 6X SSC for 10min, using the Bio-Dot microfiltration apparatus (BioRad) and following the manufacturer’s instructions. After DNA binding, the membrane was incubated in 2X SSC for 5min at RT, cross-linked for 2h at 80°C and blocked with Tropix I-block blocking reagent (Tropix) in PBS with 0.1% of Tween-20. Primary antibody against 5-methylcytosine (BI-MECY-1000, Eurogentec) was incubated o/n at 4°C and alkaline phosphatase conjugated secondary antibody was incubated for an additional 1h at RT. Dot Blot was revealed by a chemiluminiscent reagent (Tropix) and detected using HyperfilmTM enhanced chemilumincescence films (Amersham). For loading control, membrane was stained with 0.04% methylene blue for 15–30 minutes at RT.

For cell cycle analysis, cells were washed twice with phosphate-buffered saline (PBS) and resuspended in 0.2% Tween-20 in PBS and 0.5 mg/ml Rnase A (Sigma) and incubated for 30 min at 37°C. Subsequently, cells were stained with 25 μg/ml of propidium iodide (Sigma) and analyzed using a BD FACSCalibur flow cytometer (Becton Dickinson).

### Bisulfite treatment and methylation analysis

For bisulfite treatment 1 μg of genomic DNA was modified using the CpGenome modification kit (Millipore) according to the manufacturer’s recommendations. PCR fragments were amplified with specific primers using high fidelity Platinum Taq DNA polymerase (Life Technologie) according to the manufacturer’s instructions. Amplified products were cloned into pGEMT vector (Promega) and ten randomly selected clones were sequenced with SP6 and T7 primers for each gene.

### Histone extraction and western blot

OSKM-infected BJ cells were treated with the indicated concentration of CM272 compound and collected at the indicated time points after treatment. Histone extraction and chromatin immunoprecipitation were performed as described [21]. Briefly, cell pellets were resuspended in lysis buffer containing 10 mM HEPES pH 7.9, 10 mM KCl, 1.5 mM MgCl2, 0.5 mM DTT and Complete protease inhibitor cocktail (Roche), and incubated for 30 min on ice with 0.2 M HCl. Supernatants were collected, washed and subjected to dialysis twice against 0.1 M glacial acetic acid (1h each time) and three times against water for 1 hour, 3 hours and o/n, respectively. Histone concentration was determined by Bradford assay (Bio-Rad). Equal amounts of histone protein were separated by 15% SDS-polyacrylamide gel electrophoresis and transferred onto a nitrocellulose membrane (Bio-Rad). The membranes were blocked for 1 hour with Tropix I-block blocking reagent (Tropix) in 0.1% Tween-20 PBS and incubated with a monoclonal antibody against H3K9me2 (Abcam) o/n at 4°C. An antibody against total H3 (Millipore) was used to confirm equal loading of total histone extracts. Secondary antibodies coupled with alkaline phosphatase were added for an additional 1h and Immuno-reactive bands were developed using a chemiluminescent substrate (Tropix) and Hyperfilm ECL plus films (Amersham).

### Chromatin immunoprecipitation (ChIP)

Chromatin immunoprecipitation was performed as described [21]. Briefly chromatin cross-linking was carried out by adding 1% of formaldehyde and incubating at RT for 15 min. A total of 10^7^ cells were used for making one cell pellet per ChIP. BJ cells were infected with OSKM plus rtTA2M2 lentiviruses and incubated with CM272 at 200nM for 48h. For H3K9me2 ChIP analysis the chromatin was obtained prior to doxycycline induction. For ChIP of the OCT4 and SOX2 transcription factor in early reprogramming, the chromatin was obtained 48h after doxycycline induction. H3K4me3 ChIP analysis was performed 5 days after doxycycline induction. Non-treated OSKM-infected BJ cells collected at the same time points were used as controls. Cross-linking was blocked adding 0.125 M glycine solution and incubating for 5 min at RT. The cell pellet was washed with PBS before being frozen. Cell pellet was resuspended in SDS lysis buffer with protease inhibitors cocktail (Roche) and sonicated for 30s pulses at 30s intervals. DNA content of the resulting chromatin was quantified using Nanodrop and chromatin fragments were isolated using antibodies for H3K9me2 (Abcam), H3K4me3 (Abcam), OCT4 (Abcam) and SOX2 (R&D). After immunoprocipitation, DNA was washed, eluted and phenol-chloroform extracted for qPCR analysis.

### Expression array and accession numbers

For transcriptional analysis, high quality RNA samples (RQI>9) were hybridized into the GeneChip Human Gene 2.0 ST Array (Affymetrix). cDNA was prepared from 200 ng of total RNA and then fragmented and biotinylated using Affymetrix GeneChip WT PLUS Reagent Kit. Labeled sense cDNA was hybridized to the Affymetrix Human Gene 2.0 ST microarray according to the manufacturer protocols and using GeneChip Hybridization, Wash and Stain Kit. Genechips were scanned with the Affymetrix GeneChip Scanner 3000. Background correction and normalization were done using RMA (Robust Multichip Average) algorithm [25]. R and Bioconductor [26] were used for preprocessing and statistical analysis. LIMMA (Linear Models for Microarray Data) was used to find out the probe sets that showed significant differential expression between experimental conditions. Functional enrichment analysis of Gene Ontology (GO) categories was carried out using standard hypergeometric test [27]. The biological knowledge extraction was complemented using Ingenuity Pathway Analysis (Ingenuity Systems). Microarray data were deposited in NCBI GEO database with accession number GSE95307. Microarray expression and ChIP-seq data sets were obtained from the GEO database with the following accession numbers: GSE24182 [28], GSE36570 [9], GSE25970 [29], and GSE62777 [8].

### Statistical analysis

All data presented are representative of at least three independent experiments that yielded similar results. Statistical analyses were performed with the Prism software (Graphpad).

### Ethics statement

All procedures described in this work were approved by the University of Navarra Ethical Committee as well as by the Advisory Committee for Human Tissue and Cell Donation and Use, according to Spanish and EU legislation. Primary cells from healthy donors or patients were obtained after written informed consent.

## Results

### Reversible dual G9a/DNMT inhibitor lead compounds show potent *in vitro* cellular activity in human fibroblasts

**Fig 1:**
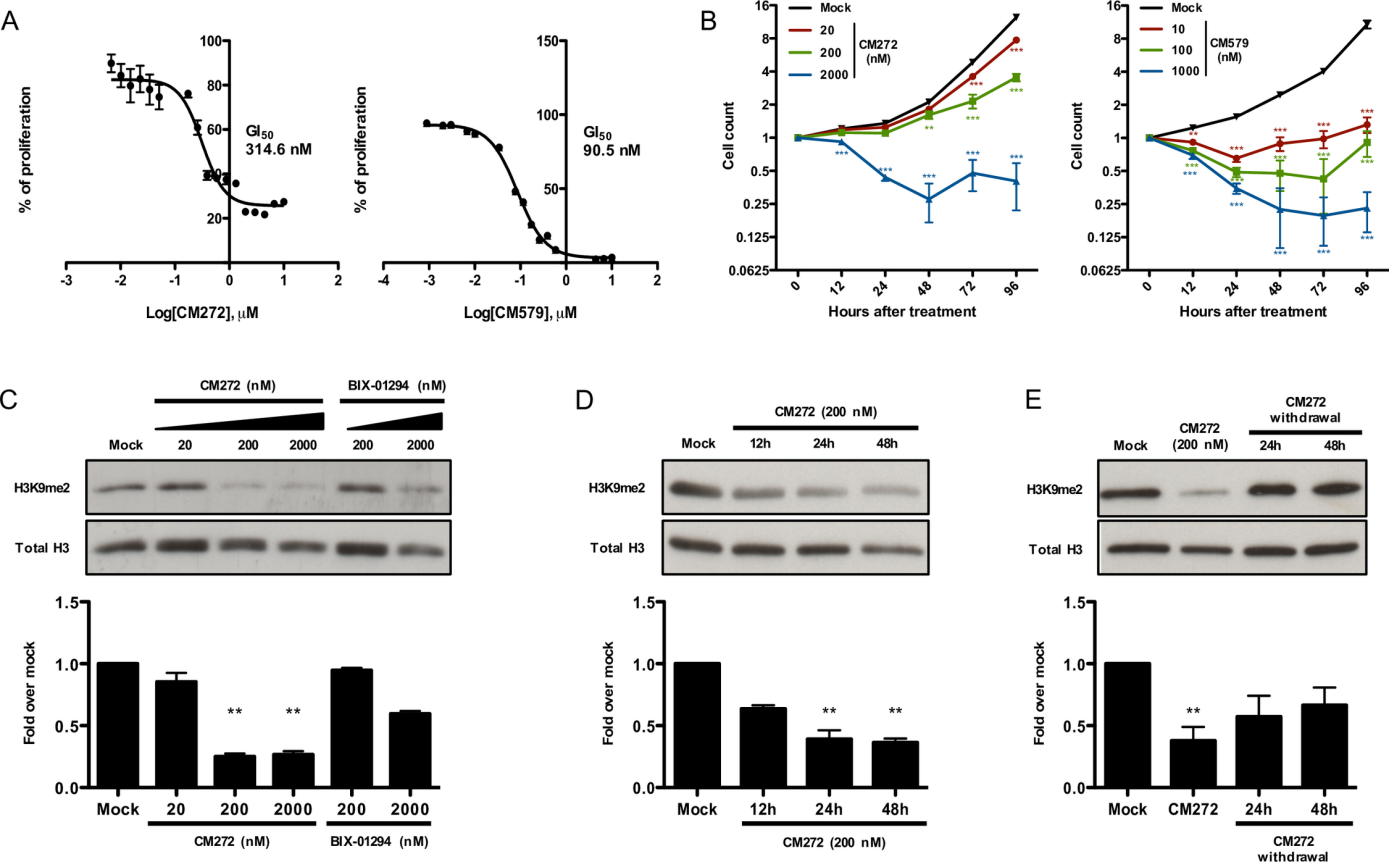
Reversible dual G9a/DNMT inhibitor activity in BJ cells. (A) Proliferation assay of OSKM-infected BJ cells measured by MTS after 48h incubation with increasing concentrations of CM272 (left) or CM579 (right). GI_50_ is denoted. (B) Cell count at different times after treatment with CM272 (left) or CM579 (right) at the indicated doses. Error bars denote SD of three independent experiments. Two-way ANOVA with Bonferroni post-tests to compare replicate means was used. (C) Analysis and quantification of H3K9me2 levels 48h after treatment of BJ cells with different doses of CM272 and BIX-01294. (D) Time course analysis and quantification of H3K9me2 levels after CM272 treatment. (E) H3K9me2 levels 24h and 48h after CM272 withdrawal. WB images are representative of at least three independent experiments. Kruskal-Wallis with Dunn’s test as a post-hoc was used. * p<0.05, ** p<0.01, *** p<0.001.

We hypothesized that our new reversible dual G9a/DNMT inhibitor lead compounds CM272 and CM579 could improve the efficiency of OSKM-based human cell reprogramming. To corroborate this hypothesis first we evaluated, by MTS (3-4(,5-dimethylthiazol-2-yl)-5-(3-carboxymethoxyphenyl)- 2-(4-sulfophenyl)-2H-tetrazolium) assay, the effect of CM272 and CM579 compounds on a human fibroblasts cell line (BJ), before reprogramming induction. We detected that the GI_50_ for CM272 and CM579 after 48h of treatment was in the nM range (CM272 GI_50_ = 314.6 nM and CM579 GI_50_ = 90.5 nM) (Fig 1A). As expected, both compounds inhibited cell proliferation, blocked cell cycle progression and induced cell death in a dose-dependent manner (Fig 1B and S1 Fig) [21]. CM579 compound dramatically reduced the number viable cells, with more than 80% of cell death at 48h after treatment with doses even 10 times lower than GI_50_. Since this effect will compromise iPSC generation, CM579 compound was discarded for reprogramming experiments and was not further analyzed. Thus, we focus on the effect of CM272 in global levels of H3K9me2 and 5’Methylcytosine (5mC). Compared to BIX-01294, a higher, transient and dose-dependent reduction of H3K9me2 levels were observed, starting to decrease 12h after CM272 addition, and restoring H3K9me2 to normal levels 48h after CM272 withdrawal (Fig 1C–1E). Furthermore, 5mC levels were also reduced (S1 Fig), indicating a reversible dual G9a/DNMT methyltransferase inhibitory activity.

### Reversible dual G9a/DNMT inhibitor increases reprogramming efficiency from primary cells

**Fig 2:**
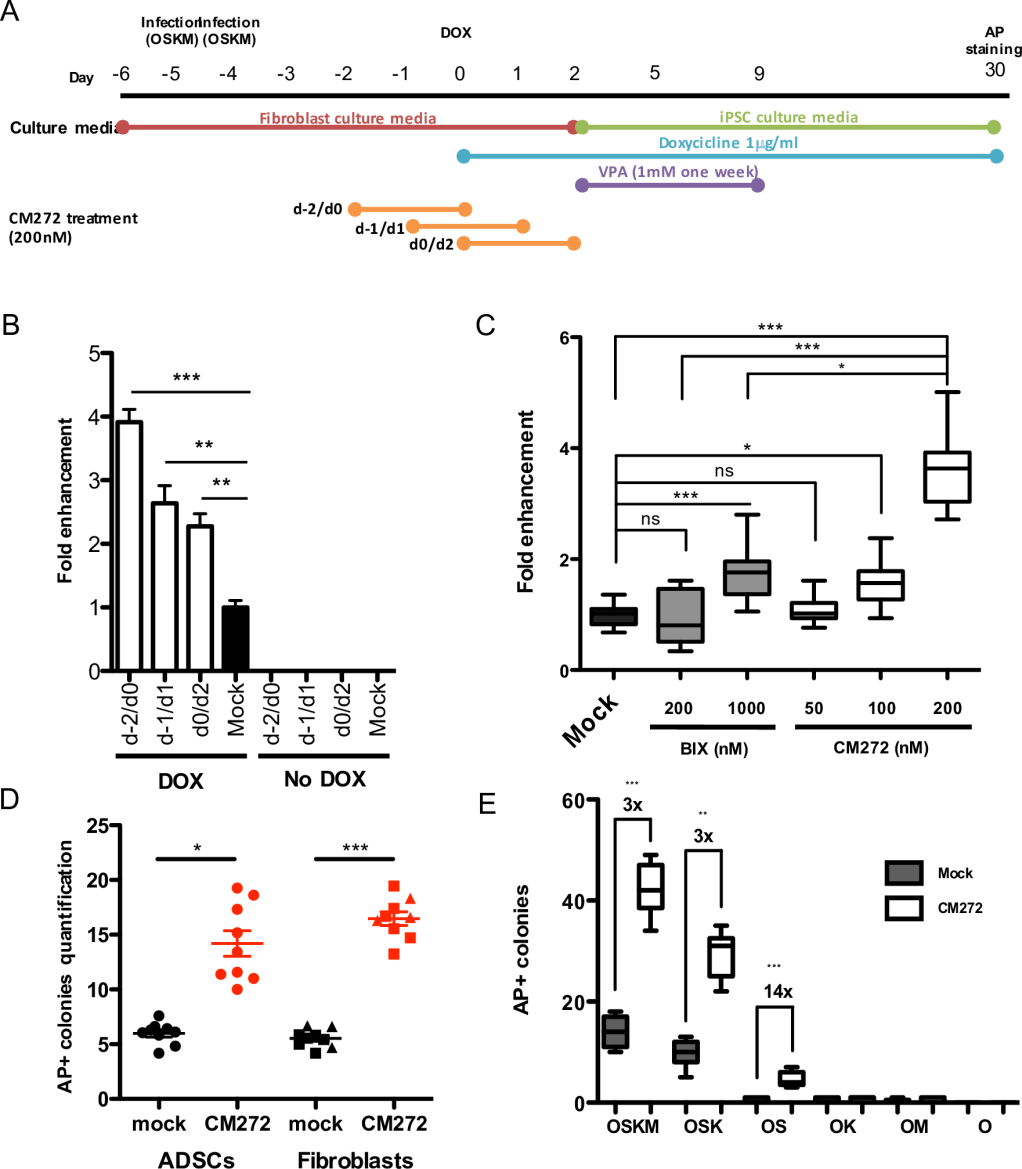
Reversible dual G9a/DNMT inhibitor activity increases reprogramming efficiency. (A) Schematic representation of the reprogramming process. (B) Quantification of AP^+^ colonies at day 30 of cell reprogramming in cells treated with CM272 at the indicated times related to doxycycline induction. Mock indicates no CM272 treatment. Error bars denote SD of three independent experiments. (C, D and E) Quantification of AP^+^ colonies at day 30 of cell reprogramming in cells treated with BIX-01294 or CM272 at the indicated doses (C), in primary cells treated with CM272 (D), and in BJ cells infected with the indicated mixtures of TFs (E). Fold increase over mock is indicated. Mock indicates no CM272 treatment. Circles ADSCs; Squares fibroblasts; Triangles PH1-fibroblasts. Kruskal-Wallis with Dunn’s test as a post-hoc was used. * p<0.05, ** p<0.01, *** p<0.001, ns: not significative.

To test whether CM272 could enhance human iPSC generation, we used a doxycycline-inducible lentiviral system for cell reprogramming that allowed us to control reprogramming initiation [24] (Fig 2A). Fibroblasts were treated at different intervals before and/or after OSKM induction with 200 nM of CM272 for 48h, condition that was showed to reduced H3K9me2 levels without compromising cell proliferation and cell cycle progression (Fig 1). Moreover, gene expression from lentiviral vectors was not affected by CM272 treatment (S2 Fig). Measuring the number of AP^+^ colonies 30 days after induction, we found that CM272 increased the reprogramming efficiency almost four-fold when added 48h before doxycycline administration (Fig 2B). The number of iPSC clones was still significantly increased when CM272 was added from the day before OSKM induction onwards, although this effect was slightly reduced. No iPSC generation was observed in the absence of OSKM induction. In comparison to BIX-01294, the effect of CM272 was higher and decreased in a dose-dependent manner (Fig 2C). Addition of CM272 did not have any effect on the time to induce cell reprograming. To test if CM272 compound was also able to enhance iPSC generation of difficult to reprogram cell types, we used the doxycycline-inducible lentiviral system to reprogram both healthy donor adipose derived mesenchymal stem cells (ADSCs) and healthy and patient-derived primary fibroblasts. In our hands, iPSC generation from ADSCs was already achieved but the efficiency was lower compared to fibroblasts [22,23]. After CM272 treatment iPSC generation was improved 2.5 and 3 folds in ADSCs and primary fibroblast respectively with no differences within different donors (Fig 2D and S2 Fig). We further test our CM272 compound during reprogramming with non-integrating vectors which would be more relevant for any clinical use. We used episomal and RNA-based systems [30,31] to reprogram BJ cell line and primary cells. In these experiments CM272 treatment was performed after reprogramming induction. Similar results were observed using episomal vectors, however, no iPSC colonies were obtained after CM272 treatment whit the RNA-based reprogramming system (that uses Sendai vectors), although this method is very efficient (S1 Table). The lack of iPSCs is probably due to the strong type I IFN response induced by CM272 [21], treatment that impairs virus infection and RNA replication [32,33]. This effect could compromise the expression of the TFs from the viral vector, completely disabling the reprogramming process. In summary, these results clearly indicate that our reversible dual G9a/DNMT activity inhibitor compound was effective on primary cells, representing a valuable advantage for some iPSC applications.

### CM272 allows iPSC generation with only two factors

Since BIX-01294 and other small molecules were able to replace some of the reprogramming factors during iPSCs derivation [14–16], we further investigated whether CM272 improved iPSC generation in the absence of some transcription factors. Lack of OCT4 totally avoid the appearance of AP^+^ colonies indicating that this factor is essential for iPSC generation under these conditions (S2 Table). In the absence of cMYC, we observed that reprogramming efficiency was still improved 3 times, although the overall number of AP^+^ colonies was slightly reduced (Fig 2E). In the absence of CM272, reprogramming with only two factors, dramatically reduced iPSC generation, with only sporadic colony formation. However, CM272 improved the generation of iPSC colonies in the presence of SOX2 and OCT4, increasing the efficiency more than 10 times (Fig 2E). In contrast, reprogramming with only two factors with BIX-01294 required the presence of RG108 or BayK [16]. In summary, although CM272 compound does not rescue the total number of iPSC colonies observed with OSKM, it improves the efficiency of iPSC generation, allowing the generation of iPSC with only 2 factors.

### iPSC generated in the presence of CM272 show pluripotent features

**Fig 3:**
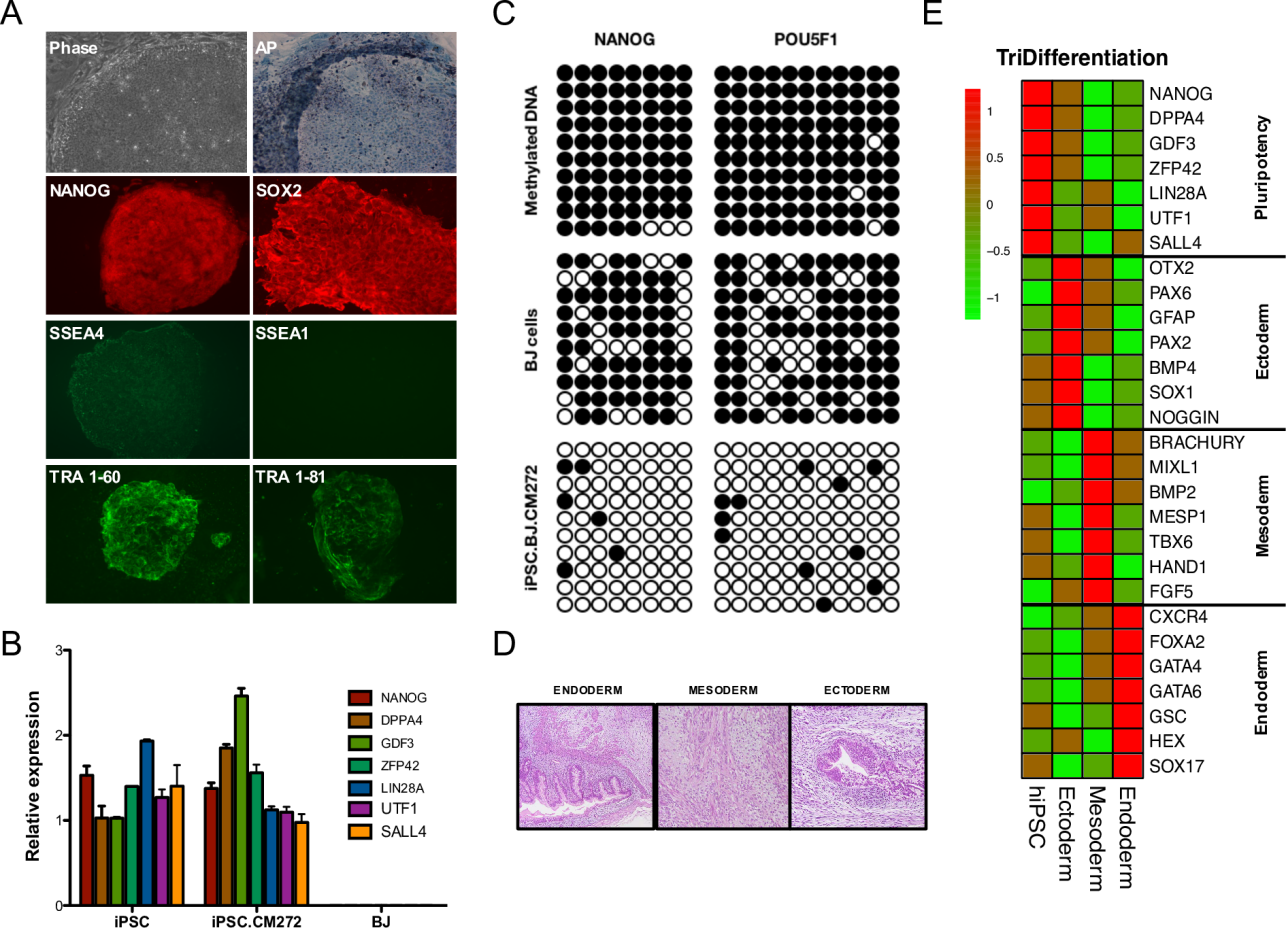
iPSCs resemble pluripotent features. (A) Representative images of the characteristic morphology of pluripotent cells, AP^+^ staining and immunofluorescence of indicated pluripotency markers in a stablished iPSC cell line generated in the presence of CM272. (B) Expression levels measured by qPCR of indicated pluripotency-associated genes in a stablished iPSC cell line generated in the presence of CM272. Error bars denote SD of three independent measurements. (C) Bisulfite sequencing of NANOG and POU5F1 (OCT4) promoter regions in a stablished iPSC cell line generated in the presence of CM272. BJ cells and methylated DNA were used as controls. (D) Hematoxylin and eosin staining shows a teratoma from an iPSC cell line generated in the presence of CM272 containing multiple tissues, including tissues from the three germ layers (ectoderm, endoderm and mesoderm). Results for other iPSC lines are summarized in S3 Table. (E) Heatmap showing principal pluripotency and lineage specific markers differentially expressed in iPSC after trilineage differentiation.

iPSCs generated in the presence CM272 were maintained in the absence of DOX for more than 20 passages, and were phenotypically and functionally similar to classic human pluripotent cells. Generated iPSCs presented the characteristic morphology of pluripotent cells, were positive for AP staining and expressed pluripotency markers (Fig 3A and 3B). Furthermore, bisulfite sequencing confirmed demethylation of the NANOG and POU5F1 (OCT4) promoters (Fig 3C). iPSCs were able to form teratomas comprising tissues of all three germ layers (Fig 3D and S3 Table), and trilineage *in vitro* differentiation dramatically reduced the expression of pluripotency markers and induced endodermal, mesodermal and ectodermal genes (Fig 3E).

### CM272 improves early reprogramming events after OSKM induction

**Fig 4:**
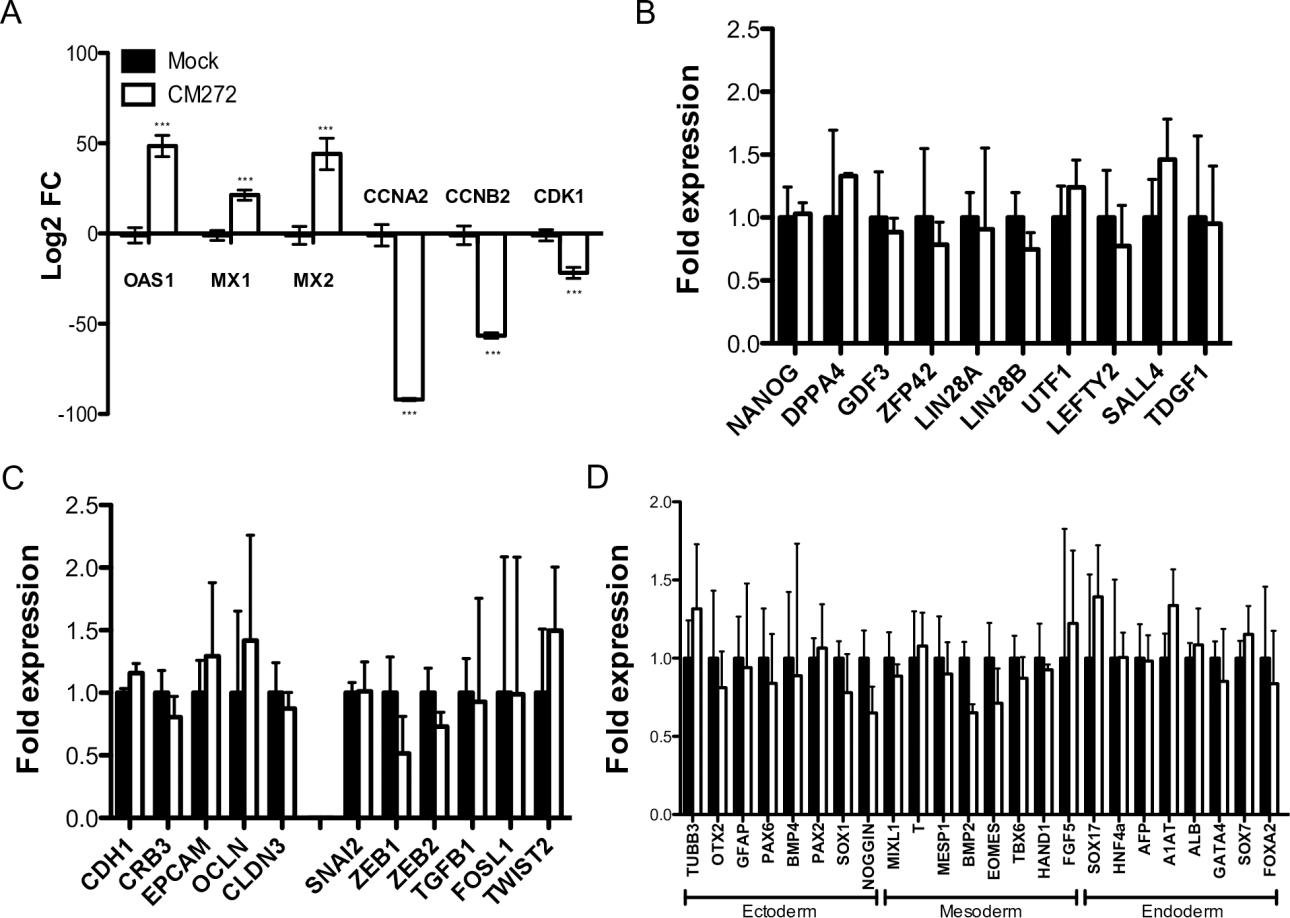
CM272 treatment does not affect specifically the reprogramming program. (A) Gene expression analysis of type I IFN response (OAS1, MX1 and MX2) and cell cycle (CCNA2, CCNB2 and CDK1) related genes after CM272 treatment. (B, C and D) Analysis of expression by qPCR of pluripotency associated genes (B), MET/EMT associated genes (C), and lineage related genes (D) after CM272 treatment. Means and SD of three independent experiments are represented. Two-way ANOVA with Bonferroni post-tests to compare replicate means was used. *** p<0.001. Not significative differences were observed in pluripotency, MET/EMT or lineage related genes.

To elucidate the mechanisms underlying improved cell reprogramming using CM272, we first analyze the gene expression profiling after treatment with CM272 compound and before treatment with doxycycline. In accordance with the proposed mechanism of action, CM272 induced the expression of type I IFN response genes (OAS1, MX1, MX2) and inhibited cell cycle related genes (CCNA2, CCNB2, CDK1) (Fig 4A and S4 Table) [21]. Then we compared gene expression changes with the major dynamic expression patterns that have been described during human reprograming, with special focus in early events during cell reprogramming [8]. We found that CM272 induced weak changes in early and late somatic categories (46/462 and 33/378 genes altered respectively, S3 Fig), and that most of these changes were found in the opposite direction to what it is expected during reprograming. Furthermore, CM272 treatment did not significantly altered the expression of key pluripotency associated genes (Fig 4B) or the expression of MET associated genes, a critical step in the reprogramming process (Fig 4C) [28,29]. Finally, the expression of genes associated to lineage conversion were also not statistically affected (Fig 4D). Taken together these results clearly indicate that CM272 by itself does not induce cell reprogramming.

**Fig 5:**
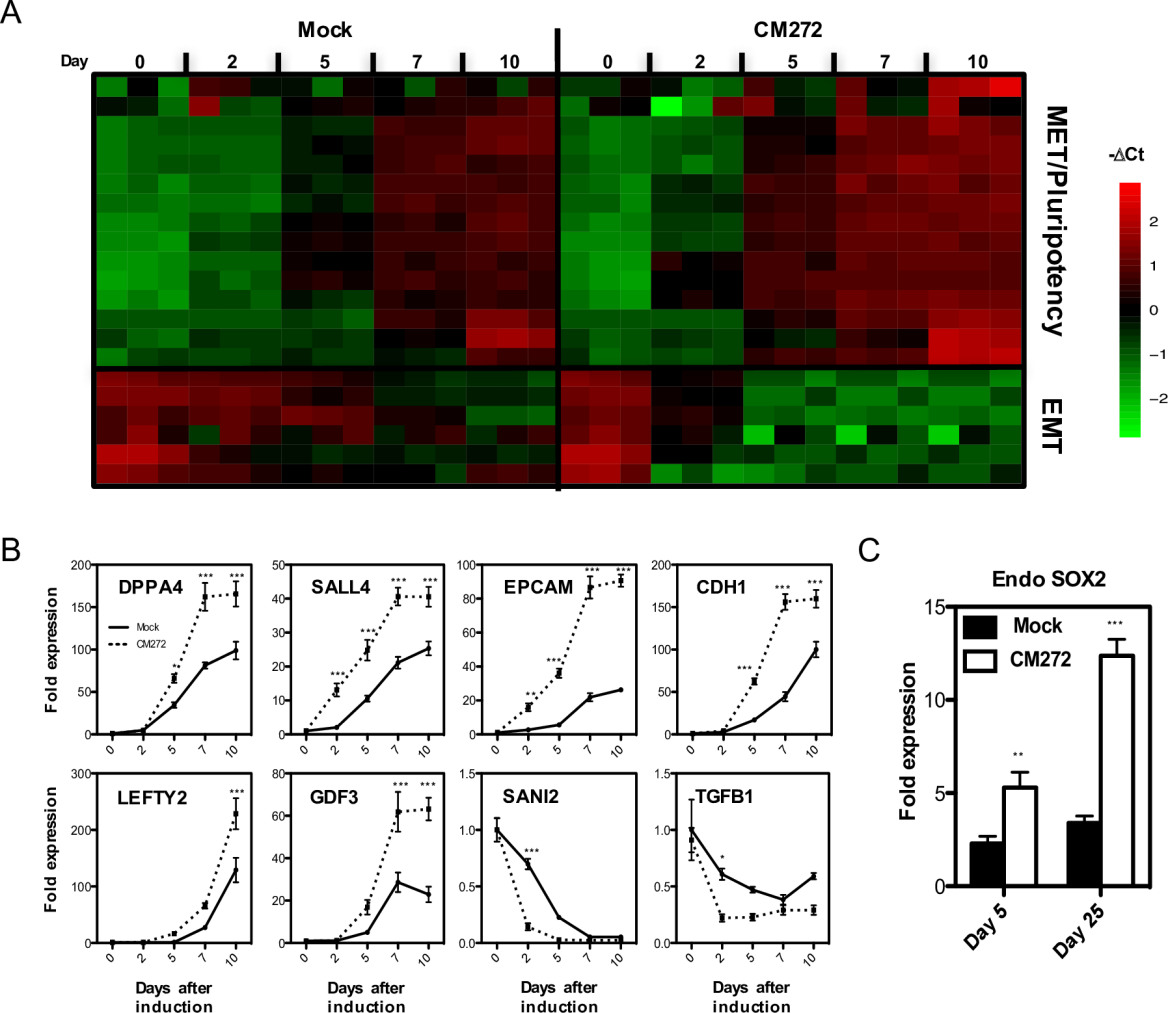
Kinetics of gene expression after OSKM induction in CM272 treated BJ cells. (A and B) Gene expression analysis of MET, EMT and pluripotency associated genes in CM272 treated cells at different time points after OSKM induction. OSKM-infected BJ cells without CM272 treatment (mock) were used as controls. (C) Endogenous SOX2 expression at the indicated times after doxycycline induction in OSKM-infected BJ cells treated with CM272. OSKM-infected BJ cells without CM272 treatment (mock) were used as controls. Means and SD of three independent experiments are represented. Two-way ANOVA with Bonferroni post-tests to compare replicate means was used. * p<0.05, ** p<0.01, *** p<0.001, ns: not significative.

We next analyzed gene expression changes induced by CM272 after OSKM induction with doxycycline. Due to the transient effect of the CM272 treatment we focused on a set of genes described to be induced early in the reprogramming process, as well as key pluripotency-associated genes (S5 Table) [8,10]. We observed that once the reprograming process was initiated, CM272 induced the expression of MET and pluripotency associated genes, whereas the expression of genes promoting EMT decreased significantly. Changes observed in gene expression were significantly higher after CM272, although the kinetics of the process was the same. Recent studies have shown that several key reprograming factors, like DPPA4 and GDF3, are located at regions refractory to OSKM-binding during the first steps of reprogramming [9]. Interestingly, the expression of these factors as well as the induction of CDH1, required for complete cell reprogramming, were also increased in the presence of CM272 (Fig 5A and 5B). The expression of endogenous SOX2, a marker of late phase initiation in cell reprogramming [5], was also upregulated after CM272 treatment (Fig 5C).

### CM272 decreases H3K9me2 repressive mark enhancing TF binding to genome

**Fig 6:**
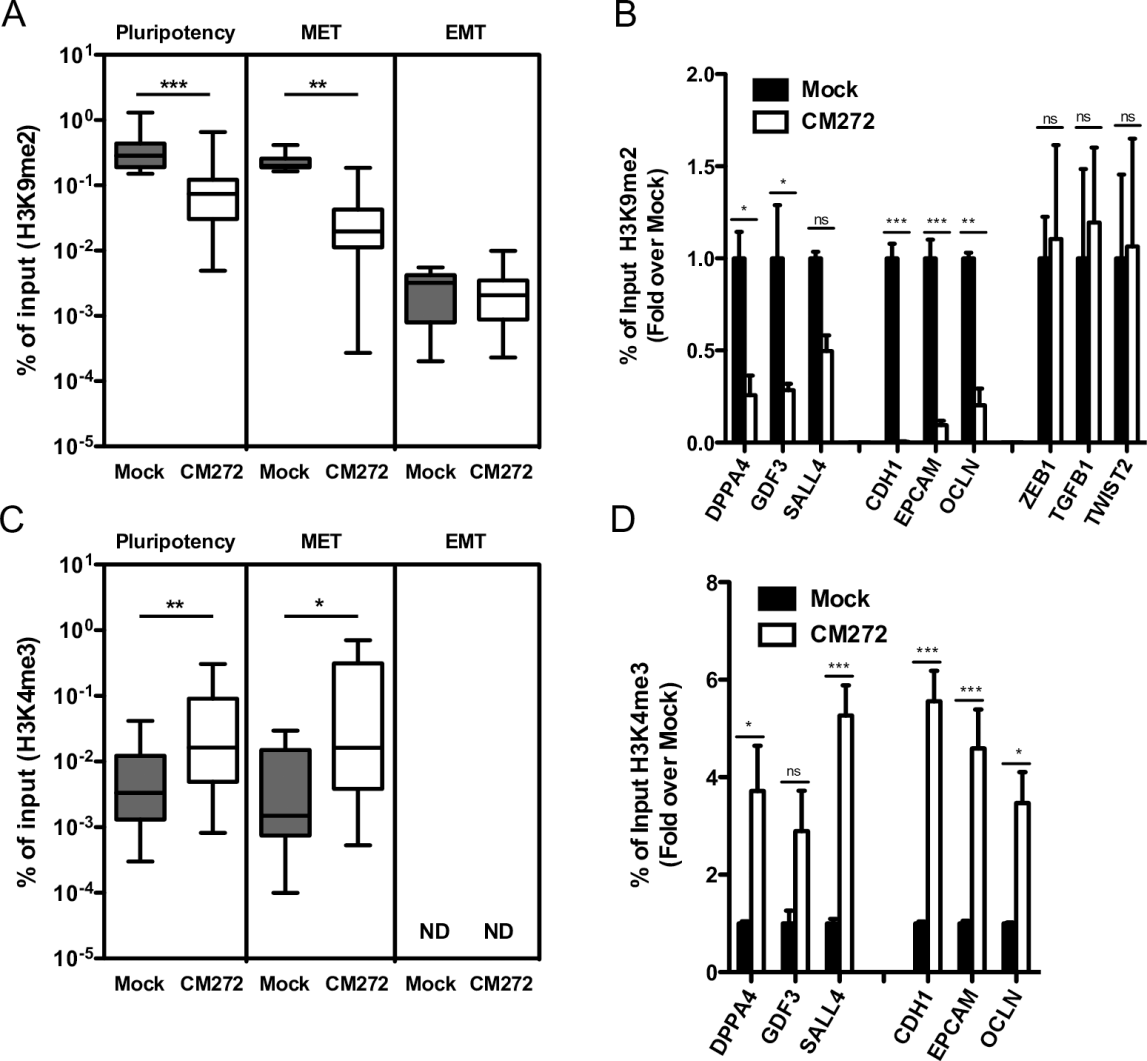
Analysis of H3K9me2 and H3K4me3 levels after CM272 treatment. ChIP-qPCR at different promoter regions of pluripotency and MET associated genes. (A and B) H3K9me2 ChIP-qPCR analysis in CM272-treated cells before OSKM induction. (C and D) H3K4me3 ChIP-qPCR analysis in CM272-treated cells 5 days after OSKM induction. Means and SD of three independent experiments are represented. ND: not-determined. Two-way ANOVA with Bonferroni post-tests to compare replicate means was used. * p<0.05, ** p<0.01, *** p<0.001, ns: not significative.

We further investigated the epigenetic signature of the same set of selected genes (S5 Table) significantly altered by CM272 during iPSC generation. After CM272 treatment and before OSKM induction we observed a decrease in H3K9me2 levels in the promoter regions of MET- and pluripotency-associated genes, indicating that the reduction of this repressive histone mark would favor an accessible chromatin conformation (Fig 6A and 6B). Decrease in H3K9me2 was also associated with an increase in the H3K4me3 activation mark five days after OSKM induction, further supporting an open conformation of the chromatin with induction of the reprogramming process (Fig 6C and 6D).

**Fig 7:**
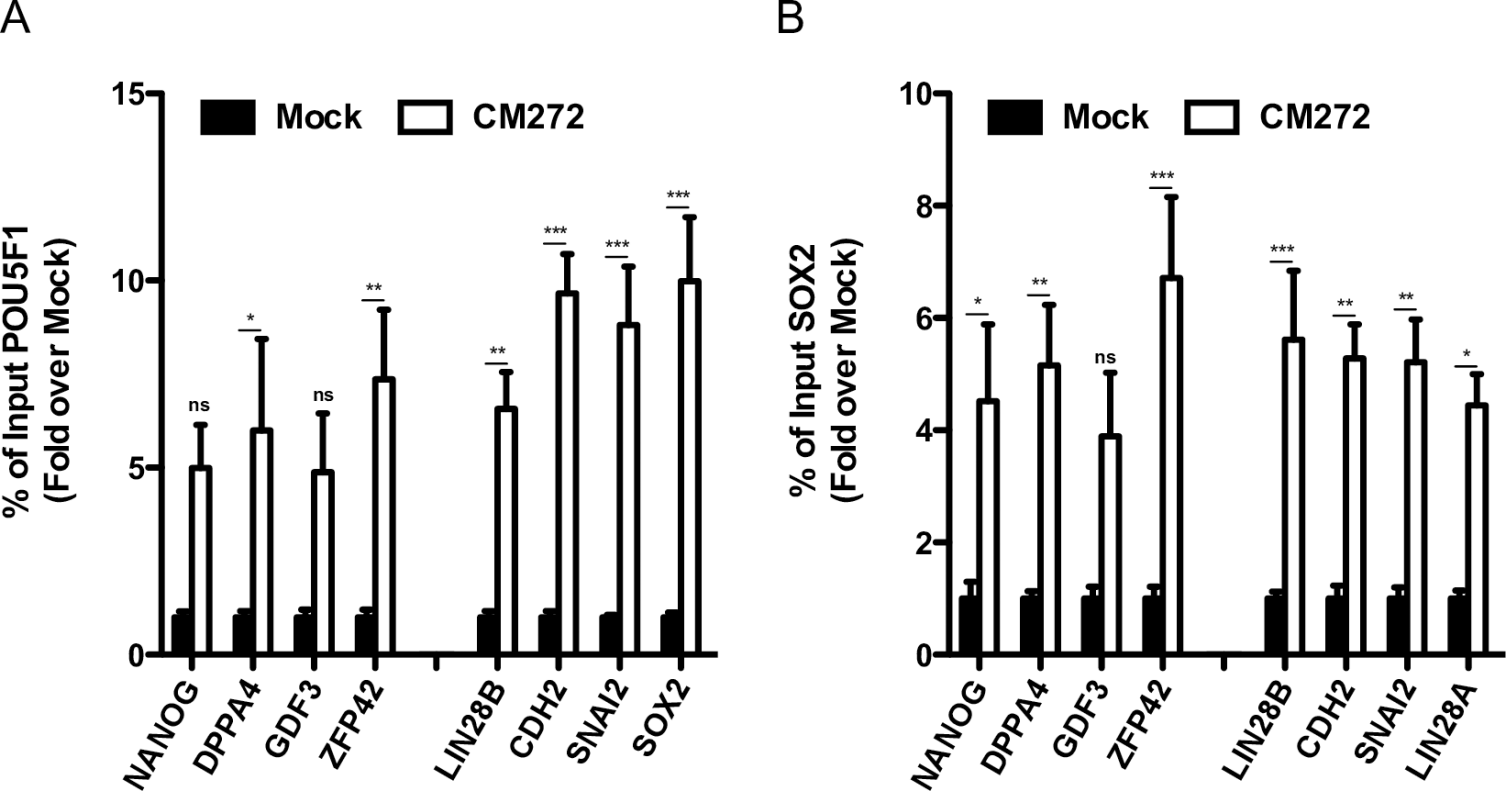
Analysis of transcription factor binding at specific promoter regions after CM272 treatment. (A) POU5F1 and (B) SOX2 binding at promoter regions of genes located at refractory OSKM-binding regions (NANOG, DPPA4, GDF3 and ZFP42) and genes associated to early reprogramming (LIN28A, CDH2, SNAI2 and SOX2) analyzed by ChIP-qPCR in CM272-treated cells 48h after OSKM induction. OSKM-infected BJ cells without CM272 treatment (mock) were used as controls. Means and SD of three independent experiments are represented. Two-way ANOVA with Bonferroni post-tests to compare replicate means was used. * p<0.05, ** p<0.01, *** p<0.001, ns: not significative.

Recent studies have shown that initial binding of OSKM to particular genomic regions is essential for pluripotency induction [9]. We further investigate how reduction of H3K9me2 repressive mark could affect the binding of the OSKM TFs to the genome during the initial phase of reprogramming. Forty-eight hours after OSKM induction, we observed significant enrichment in OCT4 and SOX2 binding at genes that promote reprogramming in CM272 treated cells, and interestingly, at the promoter regions of genes located at refractory OSKM-binding regions [9] (Fig 7A and 7B).

Taken together those results suggests that CM272 treatment improves TF engagement at promoter regions of genes involved in cell reprogramming. Since CM272 is a reversible dual G9a/DNMT inhibitor we also investigate the methylation status by pyrosequencing of the MET and pluripotency promoter regions during early phase of cell reprogramming in the presence of CM272. We were not able to detect significant changes probably due to the longer CM272 treatment required to reduce DNA methylation [21]. Together, all these results clearly indicate that CM272 treatment improves iPSC generation facilitating the engagement of reprogramming factors to the genome due to the reduction of the H3K9me2 repressive mark, and promoting the expression MET and pluripotency genes involved in cell reprogramming.

## Discussion

In this study, we showed that a new first-in-class dual and reversible G9a/DNMT inhibitor compound [21] improves reprogramming efficiency of human cells inducing heterochromatin relaxation, facilitating the engagement of transcription factors to the genome at refractory binding regions and promoting the early events during cell reprogramming. The combination of defined factors with small molecules targeting epigenetic factors is a strategy that would provide additional means to enhance optimal derivation of iPSCs for therapeutic application and regenerative medicine [3,4]. Cell reprogramming and in particular pluripotency induction is a multistep and low efficient process where specific genetic and epigenetic modifications are key steps [6,8]. Thus, is not surprising that chromatin-modifying enzymes have been described to play a key role as modulators of cell reprogramming [12,13]. Indeed, small molecules targeting epigenetic modulators has been reported to improve iPSC generation [34] being able to induce pluripotency even in the absence of reprogramming factors [35].

The use of small molecules to overcome reprogramming barriers, like G9a/GLP mediated H3K9 methylation [17,18], represents an attractive approach for safe and efficient iPSC derivation, mechanistic studies, high throughput screenings or therapeutic applications. Previous studies have demonstrated that inhibition of G9a with BIX-01294 can improve iPSC generation in mouse embryonic fibroblasts (MEFs) [15,16]. Here, we demonstrated that CM272 compound was able to improve iPSC derivation of difficult to reprogram primary human cells, like dermal fibroblasts and ADSCs. Moreover, to improve iPSC generation from primary cells could represent an advantage for some applications like disease modelling of rare diseases, where human samples are lacking and their accessibility is limited. The use of RNA-based reprogramming systems like Sendai vectors, that are very efficient inducing iPSC generation [30], may be compromised due to the strong IFN response induced by CM272 [32,33,36]. However, other reprogramming methods with non-integrating vectors (episomal vectors), that are less compromised by the IFN response, also increased the efficacy of reprograming after CM272 treatment, which would be more relevant for some applications. Small molecules are also valuable tools to reduce the number of transcription factors with oncogenic potential, like cMYC [37], required for efficient cell reprogramming. BIX-01294 can induce iPSC generation with of only two factors in neural progenitor cells (NPCs) and MEFs [15,16]. However, NPCs endogenously express *Sox2* and reprogramming of MEFs only with *Oct4* and *Klf4* required the use of BIX-01294 together with RG108, a DMNT inhibitor, or BayK, an L-calcium channel agonist. As CM272 also inhibits DNMT and inhibition of DNMT improves iPSC generation [14] it is tempting to hypothesize that the dual activity has a synergistic potential. The use of CM272 was able to facilitate the generation of iPSC in the presence of only OCT4 and SOX2, a condition that was only reported to reprogram cord blood cells, with already stem characteristics, and renal proximal tubular cells [38,39]. Nevertheless, the use of at least 2 transcription factors was required to generate iPSC, consistent with the role of epigenetic inhibitors that may change the chromatin conformation but do not induce reprogramming per se.

Our reversible dual G9a/DNMT inhibitor lead compounds CM272 and CM579 impaired cell growth even at doses below GI_50_. This effect was especially dramatic with CM579, fact that led us to discard this compound, focusing our efforts on the characterization of CM272 in cell reprogramming. At doses close to GI_50_, CM272 reduced the expression of several cyclins and CDKs, effect that could limit the efficacy for enhanced iPSC generation since induction of cell proliferation has been described to be important during the first stages of cell reprogramming [8]. However, by combining the appropriate dose and exposure time of CM272, improved iPSC generation was achieved. On the other hand CM272 induced IFN response related genes, in accordance to previous studies [40] reporting that ablation or pharmacological inactivation of G9a resulted in the conversion of fibroblasts into highly potent IFN-producing cells. Induction of IFN response has been associated with improvement of cell reprogramming [41] and potentially underlying the mechanism contributing to the efficacy of CM272. Additionally, G9a has been implicated in the maintenance of imprinted DNA methylation [42] and its inhibition could affect the imprinted status of the DLK1-DIO3 locus that is implicated in the reprogramming quality [43]. Our transcriptomic results clearly suggest that CM272 does not induce cell reprogramming directly but rather modifies the chromatin structure facilitating an open conformation by reducing the repressive marks. A similar effect has been described after inhibition of DOTL1, other chromatin-modifying enzyme involved in H3K79 methylation [12], suggesting that reduction of heterochromatin and loss of repressive marks are required for enhanced cell reprogramming.

In this work, we have focused in the mechanisms underlying the improvement of cell reprogramming with CM272. Our results indicate that CM272 promotes higher expression of MET and pluripotency associated genes involved in cell reprogramming [6,8]. These results may be consistent with stochastic process that occur during the early phase of reprogramming [44], where the initial interaction of OSKM with the genome induces MET [9]. We observed an increased induction of CDH1, OCLN or CLDN3, all of them important MET-associated genes. CM272 would be also facilitating the engagement of reprogramming factors to the genome due to the reduction of the H3K9me2 repressive mark, probably facilitating their expression in a larger number of cells. According to the studies performed by Soufi et al. [9], many genes required for reprogramming, such as NANOG, are located in heterochromatic regions (H3K9me3 and H3K9me2 rich regions) refractory to initial OSKM binding (OSKM-DBRs), and are not expressed until late in the reprograming process. Although the steps by which the OSKM-DBRs in fibroblasts become accessible to factor binding during reprogramming are not clearly understood, the reactivation of the genes located at DBRs is crucial for iPSC establishment. In our hands, CM272 treatment clearly increased OCT4 and SOX2 binding to essential pluripotency loci located in DBRs, such as NANOG, DPPA4 or GDF3, reactivating their expression and increasing the pace of reprogramming. Finally, activation of SOX2 has been described as a key event initiating the late deterministic hierarchical phase. The improved binding of OCT4 to SOX2 promoter and the higher expression of endogenous SOX2 observed after CM272 treatment would be in accordance with this model. Taken together these mechanisms would eventually improve the final reprogramming efficiency after CM272 treatment.

In summary, this study takes a closer look at the early events occurring during cell reprogramming of human cells after treatment with a G9a/DNMT reversible dual small molecule inhibitor compound. The increased understanding of the mechanism of enhanced reprogramming will provide strategies to improve its utility for applications like disease modelling. These results indicate that the reduction of repressive marks before induction of reprogramming facilitates the outcome of the process, suggesting that this strategy could be applied not only to iPSC generation, but also to other reprogramming processes, like trans-differentiation or direct reprogramming. Moreover, G9a/DNMT inhibitors could also have positive effects improving differentiation processes where chromatin status and epigenetic factors remodeling are also required.

## Supporting information

S1 Fig Reversible dual G9a/DNMT1 inhibitor activity. (A) Chemical structure of CM272 and CM579 compounds. (B) Cell cycle analysis in BJ cells treated with three different concentrations of CM272 (2, 0.2 and 0.02 μM) or CM579 (1, 0.1 and 0.01 μM) for 48 hours. (C and D) Dot-blot analysis of 5mC levels after treatment for 7 days (A) or 48h (B) of BJ cells with the indicated doses of CM272. (E and F) Quantification of dot-blot intensities from at least 4 independent experiments.
(TIF)

S2 Fig Effect of CM272 on reprogramming efficiency. (A) Representative pictures and quantification of GFP expression levels observed in doxycycline-inducible-GFP-infected BJ cells after doxycycline addition in the presence or absence of CM272. (B) Quantification of transcription factor expression levels observed in doxycycline-inducible-TF-infected BJ cells after doxycycline addition in the presence or absence of CM272. Error bars represent SD of three independent experiments. (C) Representative images of AP^+^ colonies at day 30 of cell reprogramming in primary cells treated with CM272 (200nM). Mock indicates no CM272 treatment.
(TIF)

S3 Fig CM272 treatment does not affect reprogramming program before doxycycline induction. (A) Hierarchical cluster analysis of the microarray data of OSKM infected BJ cells after CM272 treatment just before doxycycline induction. (B) Western blot of H3K9me2 levels after CM272 treatment of the three independent experiments. (C) Venn diagram of commonly differentially expressed genes between CM272-treated cells, pluripotency-associated genes and genes involved in early events in cell reprogramming. (D) Enrichment analysis at the major dynamic expression patterns during human iPSC generation of differentially expressed genes in OSKM-infected BJ cells after CM272 treatment and before doxycycline addition. (E) Differential expression (LogFC) of enriched genes of the early reprogramming events involving early and late somatic categories in the major dynamic expression patterns during human iPSC generation [8].
(TIF)

S1 Table Number of AP+ colonies and efficiency of cell reprogramming at day 30 in BJ and primary cells with the indicated reprogramming systems. (XLSX)

S2 Table Number of AP+ colonies and efficiency of cell reprogramming at day 30 in BJ cells infected with the indicated mixtures of TFs. (XLSX)

S3 Table Presence of different tissues representative of the three germ layers in teratomas from established human iPSC clones generated in the presence of CM272 compound. (XLSX)

S4 Table Gene ontology analysis of gene expression performed in OSKM-infected BJ cells treated with CM272 just before doxycycline induction. (XLSX)

S5 Table Selection of pluripotency-associated genes and genes induced at early phases of cell reprogramming according to the major dynamic expression patterns during human iPSC generation. (XLSX)

S6 Table List of primers used in this study. (XLSX)

S1 File Supplemental materials and methods. (DOCX)
