# Supplementary figures and images for: Reversible dual inhibitor against G9a and DNMT1 improves human iPSC derivation enhancing MET and facilitating transcription factor engagement to the genome
Source: PLoS One. 2017 Dec 27;12(12):e0190275. doi: 10.1371/journal.pone.0190275 (PMC5744984; doi:10.1371/journal.pone.0190275)

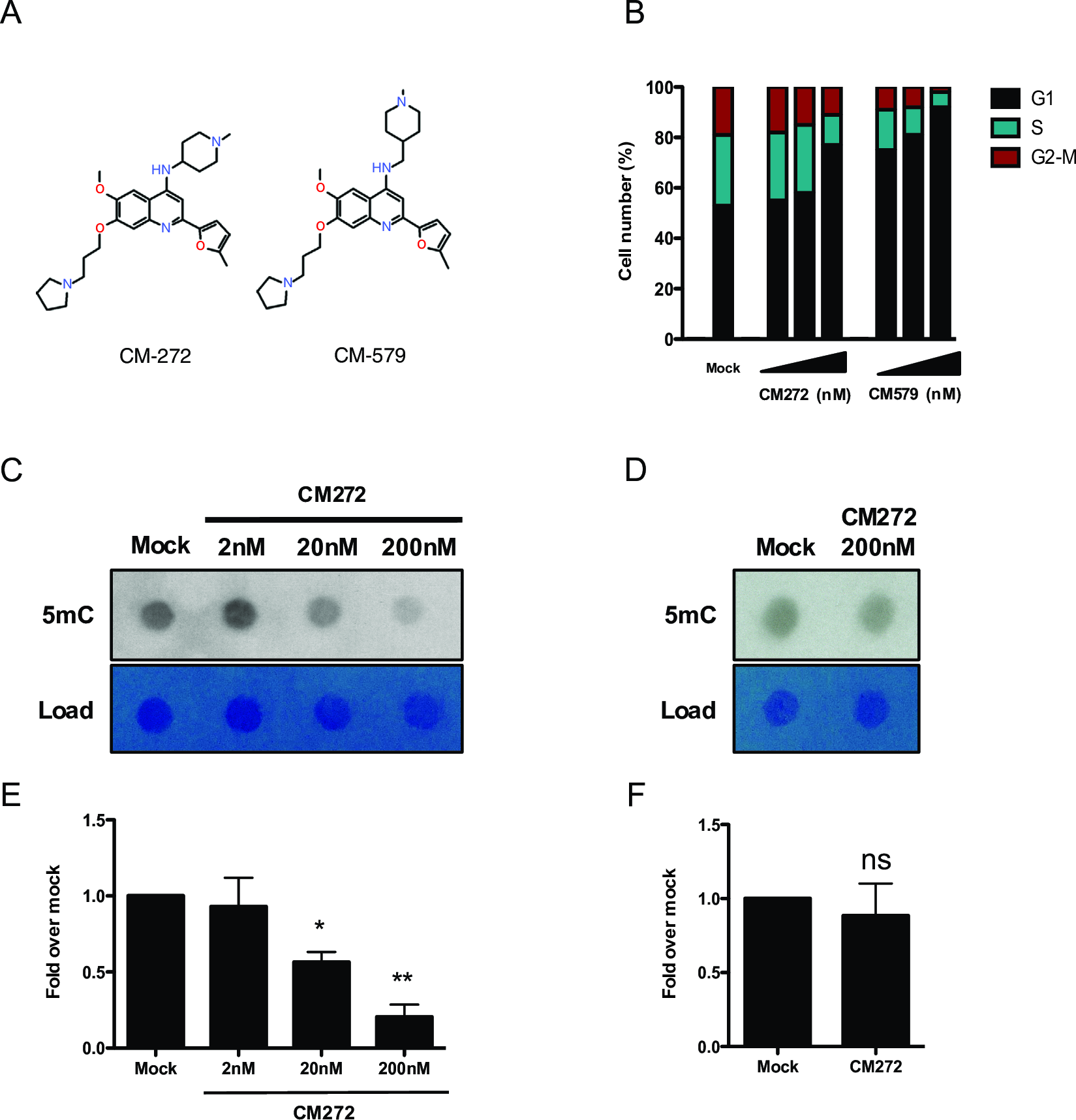

Supplement: S1 Fig — (A) Chemical structure of CM272 and CM579 compounds. (B) Cell cycle analysis in BJ cells treated with three different concentrations of CM272 (2, 0.2 and 0.02 μM) or CM579 (1, 0.1 and 0.01 μM) for 48 hours. (C and D) Dot-blot analysis of 5mC levels after treatment for 7 days (A) or 48h (B) of BJ cells with the indicated doses of CM272. (E and F) Quantification of dot-blot intensities from at least 4 independent experiments. (TIF) [file pone.0190275.s001.tif]

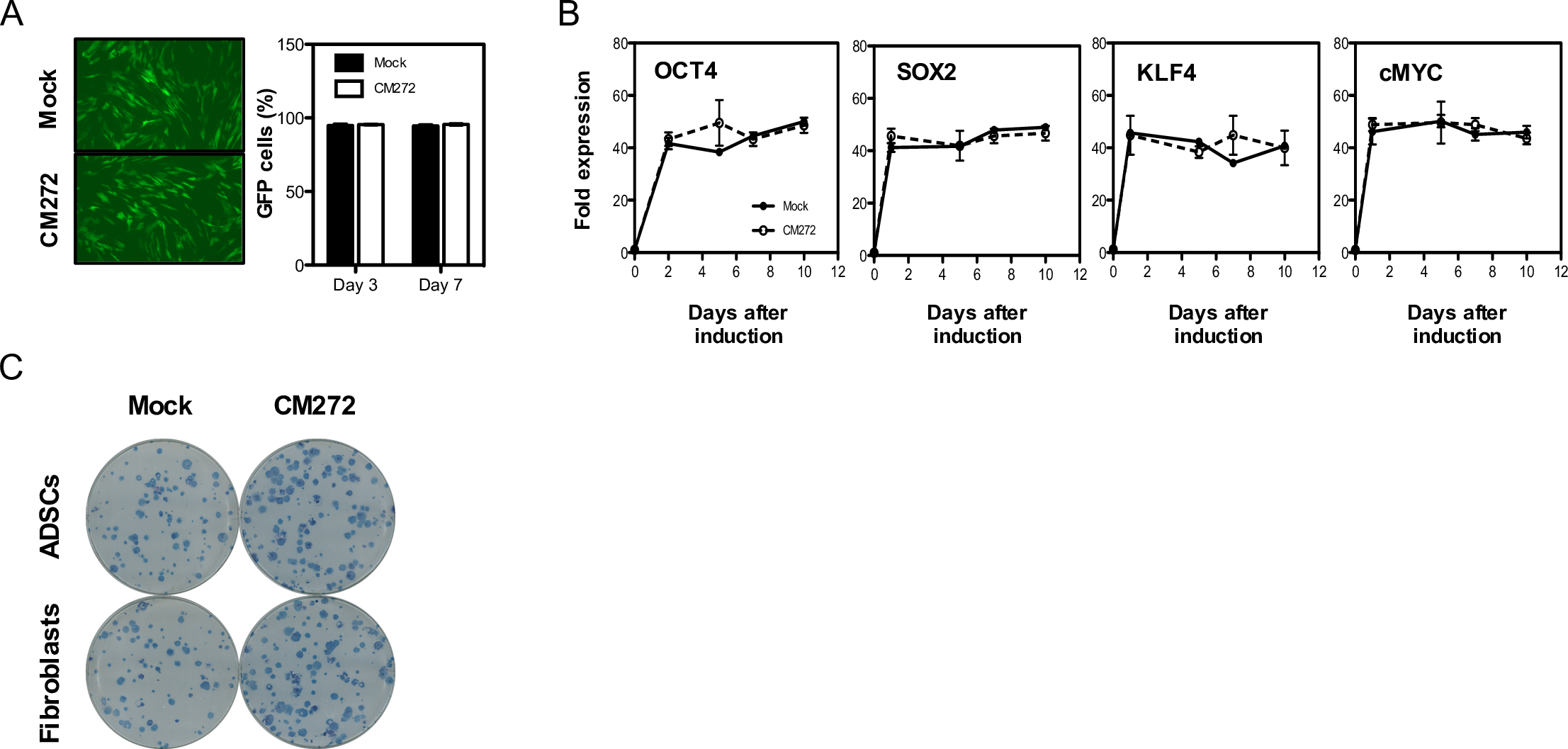

Supplement: S2 Fig — (A) Representative pictures and quantification of GFP expression levels observed in doxycycline-inducible-GFP-infected BJ cells after doxycycline addition in the presence or absence of CM272. (B) Quantification of transcription factor expression levels observed in doxycycline-inducible-TF-infected BJ cells after doxycycline addition in the presence or absence of CM272. Error bars represent SD of three independent experiments. (C) Representative images of AP+ colonies at day 30 of cell reprogramming in primary cells treated with CM272 (200nM). Mock indicates no CM272 treatment. (TIF) [file pone.0190275.s002.tif]

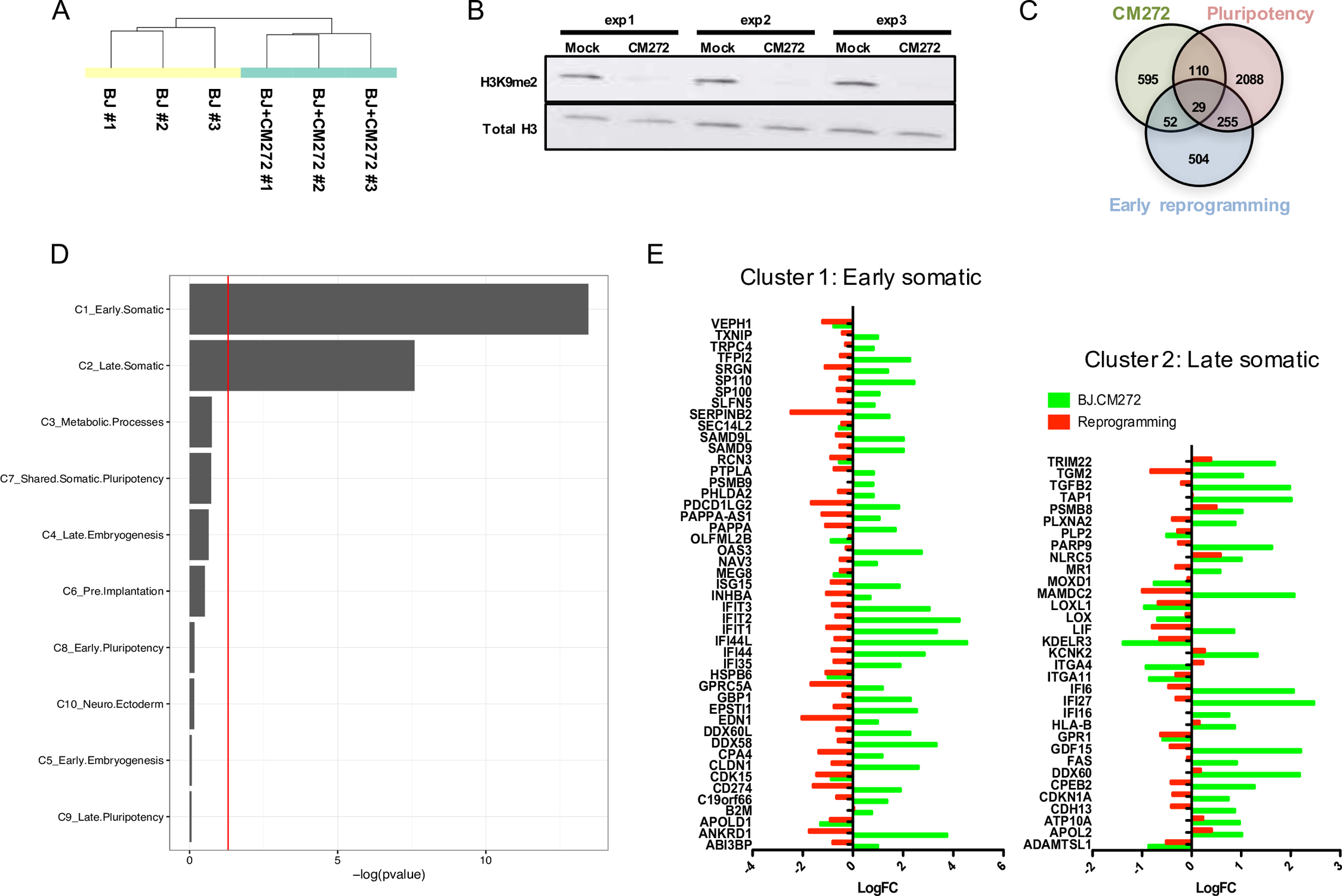

Supplement: S3 Fig — (A) Hierarchical cluster analysis of the microarray data of OSKM infected BJ cells after CM272 treatment just before doxycycline induction. (B) Western blot of H3K9me2 levels after CM272 treatment of the three independent experiments. (C) Venn diagram of commonly differentially expressed genes between CM272-treated cells, pluripotency-associated genes and genes involved in early events in cell reprogramming. (D) Enrichment analysis at the major dynamic expression patterns during human iPSC generation of differentially expressed genes in OSKM-infected BJ cells after CM272 treatment and before doxycycline addition. (E) Differential expression (LogFC) of enriched genes of the early reprogramming events involving early and late somatic categories in the major dynamic expression patterns during human iPSC generation [8]. (TIF) [file pone.0190275.s003.tif]
