# Supplementary material for: Reversible dual inhibitor against G9a and DNMT1 improves human iPSC derivation enhancing MET and facilitating transcription factor engagement to the genome
Source: PLoS One. 2017 Dec 27;12(12):e0190275. doi: 10.1371/journal.pone.0190275 (PMC5744984; doi:10.1371/journal.pone.0190275)
Supplement: S1 File — (DOCX) [file pone.0190275.s010.docx]

**SUPPLEMENTAL MATERIALS AND METHODS**

**Pyrosequencing.**

The PCR reactions for pyrosequencing were done using nested PCR with gene specific primers for the first PCR and universal biotinylated primers for the second PCR, and performed as follows (primer sequences are listed in S6 Table). After bisulfite treatment “hot start” PCR was performed with a denaturalization at 95°C for 10 minutes and for 35 cycles consisting of denaturation at 95°C for 1 min, annealing at the specific temperature for each gene for 1 min, and extension at 72°C for 1 min, followed by a final 10 min extension for all primer sets. The resulting biotinylated PCR products were immobilized to streptaviding Sepharose High Performance beads (GE healthcare, Uppsala, Sweden) and processed to yield high quality ssDNA using the PyroMark Vacuum Prep Workstation (Biotage, Uppsala, Sweden), according to the manufacturer’s instructions. The pyrosequnecing reactions were performed using the PyromarkTM ID (Biotage, Uppsala, Sweden) and sequence analysis was performed using the PyroQ-CpG analysis software (Biotage, Uppsala, Sweden). Human male genomic DNA universally methylated for all genes (Intergen Company, Purchase, NY) was used as a positive control.
